# Supplementary material for: Comparative Transcriptomics Reveal Differential Expression of Coding and Non-Coding RNAs in Clinical Strains of Mycobacterium tuberculosis
Source: Int J Mol Sci. 2024 Dec 30;26(1):217. doi: 10.3390/ijms26010217 (PMC11720245; doi:10.3390/ijms26010217)
Supplement: Supplementary file 1 [file ijms-26-00217-s001.zip › ijms-3328341- supplementary.pdf]

Supplementary Table S1: Differentially expressed sRNAs between Beijing vs H37Rv strains (*q*<0.05)

Transcription Start	Transcription Stop	Product	Expression H37Rv	Expression Beijing	qValue H37Rv vs Beijing	Transcript Size
778934	778968	predicted RNA	0	67	0	34
786950	786962	predicted RNA	0	113	0	12
1131426	1131445	predicted RNA	0	60	0	19
1508930	1508951	predicted RNA	0	60	0	21
1640667	1640685	predicted RNA	0	51	0	18
1761615	1761656	predicted RNA	139	3	0	41
1915134	1915158	predicted RNA	0	46	0	24
1951759	1951768	predicted RNA	48	0	0	9
2142097	2142154	predicted RNA	0	41	0	57
2144890	2144903	predicted RNA	0	77	0	13
2228628	2228661	predicted RNA	0	67	0	33
2234920	2234961	predicted RNA	0	60	0	41
2238106	2238148	predicted RNA	74	1	0	42
2651977	2651988	predicted RNA	2	109	0	11
3039811	3039828	predicted RNA	0	75	0	17
3315151	3315169	predicted RNA	0	60	0	18
3331717	3331755	predicted RNA	0	56	0	38
3332765	3332777	predicted RNA	0	51	0	12
3430278	3430291	predicted RNA	0	72	0	13
3548934	3548947	predicted RNA	0	43	0	13
3614415	3614453	predicted RNA	0	55	0	38
3858420	3858438	predicted RNA	0	97	0	18
3966049	3966062	predicted RNA	0	57	0	13
4035906	4035920	predicted RNA	0	43	0	14
4197334	4197374	predicted RNA	117	3	0	40
4230283	4230323	predicted RNA	0	57	0	40
4235480	4235528	predicted RNA	152	3	0	48
4312921	4312942	predicted RNA	0	92	0	21
989899	989938	predicted RNA	2	64	4,7E-247	39
51816	51827	predicted RNA	80	2	4,7E-217	11
4195981	4195991	predicted RNA	3	63	1,2E-213	10
1320007	1320040	predicted RNA	256	9	7,8E-203	33
1359451	1359468	predicted RNA	9	220	5,9E-166	17
3464483	3464543	predicted RNA	51	2	2,1E-142	60
2156666	2156697	predicted RNA	3	74	2,7E-106	31
289043	289062	predicted RNA	109	6	9,14E-96	19
4372911	4372946	predicted RNA	48	2	2,81E-91	35

2985265	2985292	predicted RNA	98	5	6,05E-90	27
3126191	3126249	predicted RNA	56	3	2,2E-83	58
1069781	1069833	predicted RNA	74	4	1,15E-72	52
3721387	3721426	predicted RNA	192	11	1,21E-71	39
3708488	3708608	predicted RNA	1	29	1,46E-62	120
133768	133947	predicted RNA	50	576	6,94E-52	179
2945020	2945068	predicted RNA	35	537	2,31E-50	48
1101784	1101806	predicted RNA	81	6	5,2E-46	22
4401849	4401904	predicted RNA	86	6	1,18E-44	55
2801195	2801241	predicted RNA	104	7	2,44E-44	46
1547580	1547622	predicted RNA	87	6	3,81E-43	42
2981838	2981861	predicted RNA	4	56	2,21E-42	23
186662	186757	predicted RNA	79	6	8,68E-40	95
1986687	1986816	predicted RNA	82	6	1,17E-34	129
1718703	1718724	predicted RNA	141	11	7,25E-31	21
1274773	1274818	predicted RNA	3167	464	9,3E-28	45
3769226	3769280	predicted RNA	184	17	1,91E-26	54
87131	87176	predicted RNA	172	17	5,59E-22	45
223529	223561	predicted RNA	151	15	2,15E-21	32
3850349	3850551	predicted RNA	33	3	5,96E-21	202
1201683	1201721	predicted RNA	8	84	1,05E-20	38
3885134	3885153	predicted RNA	81	8	3E-19	19
2585917	2585926	predicted RNA	66	7	3,12E-19	9
1043261	1043302	predicted RNA	142	14	5,2E-19	41
2430100	2430125	predicted RNA	142	15	1,84E-18	25
1783236	1783315	predicted RNA	88	9	3,17E-18	79
1365249	1365274	predicted RNA	135	15	7,84E-17	25
4046359	4046383	predicted RNA	60	6	1,31E-16	24
4117398	4117435	predicted RNA	86	10	2,66E-15	37
476662	476681	predicted RNA	18	165	3,97E-15	19
1825936	1825951	predicted RNA	6	46	1,03E-14	15
462096	462135	predicted RNA	6	53	1,87E-13	39
3347885	3347991	predicted RNA	2	21	3,29E-13	106
2987626	2987667	predicted RNA	66	8	5,59E-13	41
1967645	1967679	predicted RNA	84	10	9,79E-13	34
4393252	4393626	predicted RNA	108	16	1,03E-12	374
1178516	1178741	predicted RNA	45	5	1,28E-12	225
1733557	1733608	predicted RNA	134	17	3,33E-12	51
4137298	4137381	predicted RNA	20	171	7,87E-12	83
3907530	3907720	predicted RNA	7949	1163	2,13E-11	190
3670021	3670030	predicted RNA	98	14	7,06E-11	9
2396847	2396907	predicted RNA	769	136	1,25E-10	60
3313702	3313773	predicted RNA	66	8	1,26E-10	71
3827146	3827164	predicted RNA	214	28	3,1E-10	18

2358220	2358274	predicted RNA	274	40	5,05E-10	54
2449971	2450000	predicted RNA	142	19	6,19E-10	29
3095030	3095075	predicted RNA	7	57	8,29E-10	45
1090332	1090376	predicted RNA	825	141	8,53E-10	44
2023440	2023453	predicted RNA	4123	828	1,09E-09	13
3538374	3538396	predicted RNA	103	14	1,1E-09	22
2953050	2953418	predicted RNA	41	197	1,32E-09	368
1782592	1782764	predicted RNA	353	72	1,57E-09	172
4362154	4362209	predicted RNA	495	82	2,17E-09	55
1673402	1673445	predicted RNA	217	32	2,36E-09	43
1241396	1241605	predicted RNA	16	114	2,58E-09	209
4069234	4069252	predicted RNA	132	19	4,45E-09	18
888640	889074	predicted RNA	86	325	5,33E-09	434
1196119	1196137	predicted RNA	12	88	1,12E-08	18
2960043	2960103	predicted RNA	12	88	1,78E-08	60
3633620	3633671	predicted RNA	77	11	2,3E-08	51
1094361	1094658	predicted RNA	15	94	2,9E-08	297
2931656	2931691	predicted RNA	66	10	3,16E-08	35
1023988	1024016	predicted RNA	57	8	3,45E-08	28
2554886	2554924	predicted RNA	66	10	4,35E-08	38
794594	794636	predicted RNA	128	19	9,23E-08	42
361196	361260	predicted RNA	322	56	1,08E-07	64
580599	580806	predicted RNA	358	86	1,17E-07	207
434721	434830	predicted RNA	74	11	1,51E-07	109
3650107	3650171	predicted RNA	180	29	1,63E-07	64
1692722	1692742	predicted RNA	131	20	2,31E-07	20
1376842	1376884	predicted RNA	12	83	3,17E-07	42
1081713	1081736	predicted RNA	8	51	4,35E-07	23
3942363	3943033	predicted RNA	216	56	4,71E-07	670
1313386	1313457	predicted RNA	787	188	8,19E-07	71
830027	830064	predicted RNA	42	276	8,87E-07	37
3820583	3820832	predicted RNA	73	287	9,43E-07	249
3166595	3166693	predicted RNA	28	178	9,51E-07	98
2346189	2346204	predicted RNA	11	70	1,08E-06	15
3074566	3074605	predicted RNA	89	14	1,14E-06	39
1051503	1051547	predicted RNA	81	13	1,52E-06	44
2819881	2819897	predicted RNA	18	114	1,93E-06	16
1678916	1678947	predicted RNA	145	24	3,78E-06	31
876576	876617	predicted RNA	116	20	3,98E-06	41
1658943	1658985	predicted RNA	352	67	8E-06	42
907320	907340	predicted RNA	56	9	8,89E-06	20
1746913	1746924	predicted RNA	252	43	9,42E-06	11
183491	183537	predicted RNA	47	8	1,25E-05	46
3796542	3796594	predicted RNA	155	715	1,67E-05	52

4093058	4093810	predicted RNA	337	97	2,03E-05	752
241298	241335	predicted RNA	108	19	2,35E-05	37
4021563	4021583	predicted RNA	100	18	2,4E-05	20
2854929	2854947	predicted RNA	105	19	2,43E-05	18
34051	34152	predicted RNA	315	73	3,23E-05	101
51125	51178	predicted RNA	394	1297	4,65E-05	53
1210545	1210599	predicted RNA	138	26	5,22E-05	54
2344233	2344258	predicted RNA	91	17	5,38E-05	25
3626632	3626641	predicted RNA	142	25	5,86E-05	9
2424911	2424931	predicted RNA	113	21	5,96E-05	20
3493449	3494656	predicted RNA	101	298	6,38E-05	1207
2200640	2200732	predicted RNA	38	7	7,1E-05	92
1034802	1034906	predicted RNA	66	12	7,12E-05	104
960155	960344	predicted RNA	33	153	8,68E-05	189
2055375	2055665	predicted RNA	324	884	8,86E-05	290
565484	565747	predicted RNA	125	369	9,67E-05	263
4214119	4214153	predicted RNA	324	66	9,99E-05	34
3594366	3594430	predicted RNA	72	14	0,000128	64
841208	841230	predicted RNA	103	20	0,000142	22
3805006	3805026	predicted RNA	17	89	0,000162	20
2930018	2930078	predicted RNA	54	10	0,000165	60
4088409	4088448	predicted RNA	20	113	0,000175	39
914247	914259	predicted RNA	48	10	0,000176	12
538552	538580	predicted RNA	40	216	0,000222	28
1109211	1109275	predicted RNA	114	23	0,000261	64
635879	635937	predicted RNA	83	17	0,000262	58
2562497	2562506	predicted RNA	18	92	0,000265	9
3690950	3691306	predicted RNA	310	100	0,000278	356
2646684	2646705	predicted RNA	24	127	0,0003	21
3943975	3944017	predicted RNA	104	21	0,000345	42
528320	528610	predicted RNA	540	177	0,000367	290
1607979	1608041	predicted RNA	777	230	0,000372	62
1655550	1655614	predicted RNA	66	13	0,000461	64
2367664	2367711	predicted RNA	17	93	0,000479	47
1365844	1365864	predicted RNA	18	93	0,000495	20
456252	456268	predicted RNA	1148	278	0,000506	16
4174961	4175050	predicted RNA	59	12	0,000534	89
4163897	4163913	predicted RNA	133	27	0,000583	16
3995879	3995982	predicted RNA	437	1120	0,000597	103
541914	542144	predicted RNA	85	258	0,000667	230
1333865	1333917	predicted RNA	93	20	0,000674	52
2881713	2881767	predicted RNA	16	85	0,000686	54
1712075	1712247	predicted RNA	4	21	0,000707	172
2880049	2880084	predicted RNA	120	25	0,000718	35

1019987	1020038	predicted RNA	124	26	0,000729	51
1995850	1996158	predicted RNA	21	89	0,000845	308
1855482	1855524	predicted RNA	67	13	0,000927	42
66858	66902	predicted RNA	66	13	0,000973	44
1992585	1993159	predicted RNA	22	5	0,001033	574
2123629	2123690	predicted RNA	19	95	0,001033	61
3778373	3778738	predicted RNA	97	255	0,001062	365
1127033	1127069	predicted RNA	91	19	0,001078	36
1021648	1021689	predicted RNA	259	59	0,001091	41
2610432	2610846	predicted RNA	73	197	0,001128	414
854161	854221	predicted RNA	137	31	0,001144	60
2894448	2894490	predicted RNA	127	28	0,001148	42
3873585	3873596	predicted RNA	66	14	0,001169	11
2056213	2056505	predicted RNA	1283	4181	0,001299	292
29630	29675	predicted RNA	415	106	0,001327	45
3890913	3891008	predicted RNA	66	266	0,001434	95
1613150	1613274	predicted RNA	8	1	0,001556	124
3202031	3202429	predicted RNA	245	85	0,00156	398
3583765	3583796	predicted RNA	26	127	0,001586	31
2431432	2431475	predicted RNA	139	563	0,001662	43
3121841	3121884	predicted RNA	139	563	0,001662	43
846128	846161	predicted RNA	66	14	0,001831	33
3991705	3991799	predicted RNA	68	15	0,001864	94
3317779	3317793	predicted RNA	65	14	0,001899	14
1215156	1215521	predicted RNA	55	14	0,001979	365
4359961	4360376	predicted RNA	79	24	0,002115	415
2219260	2219761	predicted RNA	150	52	0,002235	501
2712887	2712900	predicted RNA	112	25	0,002291	13
3831734	3831904	predicted RNA	15	64	0,002291	170
1543265	1543308	predicted RNA	143	563	0,002315	43
4409077	4409141	predicted RNA	48	214	0,002345	64
3638749	3638806	predicted RNA	557	172	0,002532	57
1742201	1742217	predicted RNA	294	68	0,002639	16
3310337	3310723	predicted RNA	147	51	0,002639	386
876394	876408	predicted RNA	24	109	0,002696	14
3542043	3542134	predicted RNA	18	85	0,002866	91
978207	978458	predicted RNA	11	51	0,002918	251
2626621	2626637	predicted RNA	195	44	0,002957	16
3089010	3089033	predicted RNA	113	25	0,002957	23
3905875	3905942	predicted RNA	159	39	0,003039	67
3085567	3085586	predicted RNA	18	84	0,00305	19
626444	626459	predicted RNA	72	16	0,003187	15
1989017	1989048	predicted RNA	145	592	0,00325	31
3628061	3628094	predicted RNA	24	112	0,003329	33

3082828	3082883	predicted RNA	82	19	0,003389	55
1579587	1579603	predicted RNA	156	35	0,003436	16
2124840	2125346	predicted RNA	137	265	0,003457	506
1227571	1227600	predicted RNA	124	29	0,003754	29
130539	130716	predicted RNA	17	74	0,00406	177
1179220	1179400	predicted RNA	64	206	0,004219	180
1773848	1773933	predicted RNA	1762	656	0,004219	85
2635603	2635634	predicted RNA	146	579	0,004938	31
3551042	3551277	predicted RNA	77	212	0,005373	235
2756758	2756802	predicted RNA	105	25	0,005559	44
2832676	2832699	predicted RNA	18	79	0,006157	23
802482	802530	predicted RNA	254	774	0,006186	48
414239	414252	predicted RNA	108	26	0,006193	13
1243510	1243711	predicted RNA	105	30	0,007016	201
3613601	3613631	predicted RNA	115	28	0,007126	30
2583802	2583850	predicted RNA	123	31	0,007162	48
1679179	1679327	predicted RNA	8	36	0,008856	148
2313847	2314094	predicted RNA	11	48	0,009356	247
219380	219403	predicted RNA	84	20	0,009412	23
3132798	3132844	predicted RNA	14	60	0,009412	46
3431313	3431349	predicted RNA	29	126	0,009609	36
3994941	3995008	predicted RNA	277	84	0,009888	67
902099	902113	predicted RNA	103	26	0,010212	14
2512309	2512323	predicted RNA	89	22	0,010626	14
1348675	1348687	predicted RNA	15	63	0,010876	12
1467309	1467320	predicted RNA	18	74	0,011036	11
1264134	1264318	predicted RNA	62	17	0,011654	184
2566561	2566776	predicted RNA	147	51	0,011861	215
4209055	4209095	predicted RNA	81	20	0,012048	40
1907328	1907600	predicted RNA	102	34	0,012321	272
3387013	3387085	predicted RNA	136	37	0,012327	72
2995956	2996114	predicted RNA	123	312	0,012784	158
3232645	3232866	predicted RNA	74	196	0,012888	221
2957443	2957577	predicted RNA	190	448	0,013336	134
2026406	2026468	predicted RNA	43	173	0,013522	62
3846777	3847283	predicted RNA	134	271	0,013718	506
1144472	1144567	predicted RNA	299	103	0,013805	95
1151731	1151775	predicted RNA	135	36	0,013937	44
1577575	1577615	predicted RNA	37	153	0,014087	40
4118870	4118925	predicted RNA	29	121	0,015134	55
444202	444222	predicted RNA	531	154	0,015783	20
2223198	2223312	predicted RNA	20	82	0,015915	114
3805756	3805800	predicted RNA	61	15	0,01619	44
612910	613040	predicted RNA	6092	1770	0,016298	130

1533669	1533685	predicted RNA	124	31	0,016515	16
639960	640171	predicted RNA	110	36	0,018254	211
3953515	3953578	predicted RNA	254	79	0,018999	63
4150090	4150207	predicted RNA	86	25	0,018999	117
2498761	2498839	predicted RNA	36	137	0,019374	78
2010563	2010593	predicted RNA	152	41	0,019506	30
1211500	1211521	predicted RNA	12	49	0,01962	21
4326153	4326169	predicted RNA	28	112	0,01984	16
1205848	1205988	predicted RNA	94	255	0,020477	140
2432940	2432958	predicted RNA	66	17	0,020713	18
2926154	2926199	predicted RNA	18	75	0,020713	45
3406227	3406265	predicted RNA	34	135	0,021502	38
3418660	3418722	predicted RNA	143	41	0,0225	62
1644267	1644368	predicted RNA	267	597	0,022528	101
3939739	3939795	predicted RNA	35	139	0,022671	56
155598	155616	predicted RNA	48	12	0,022828	18
968315	968409	predicted RNA	32	120	0,023163	94
439762	439866	predicted RNA	67	19	0,023373	104
436771	436825	predicted RNA	194	59	0,02428	54
419609	419788	predicted RNA	661	278	0,02463	179
1862261	1862287	predicted RNA	158	552	0,024755	26
2580010	2580037	predicted RNA	143	40	0,024864	27
3878724	3878754	predicted RNA	36	142	0,024878	30
2062695	2062757	predicted RNA	215	68	0,025048	62
4116099	4116125	predicted RNA	121	33	0,025202	26
2096630	2096880	predicted RNA	143	302	0,025318	250
3445986	3446036	predicted RNA	1133	463	0,02656	50
4069334	4069353	predicted RNA	196	55	0,026774	19
2709980	2710055	predicted RNA	10	42	0,027561	75
301674	301712	predicted RNA	234	70	0,027921	38
934146	934162	predicted RNA	15	57	0,027921	16
1261385	1261926	predicted RNA	162	67	0,028159	541
3919380	3919398	predicted RNA	16	62	0,028274	18
1057982	1058089	predicted RNA	493	203	0,029389	107
3952678	3952722	predicted RNA	257	81	0,029389	44
561821	561856	predicted RNA	110	31	0,029476	35
2250983	2251001	predicted RNA	382	1197	0,029476	18
780420	780450	predicted RNA	33	128	0,029613	30
3162244	3162277	predicted RNA	18	72	0,029613	33
4270516	4270543	predicted RNA	127	36	0,030145	27
1168562	1168639	predicted RNA	116	340	0,030283	77
3051528	3051555	predicted RNA	128	36	0,030283	27
920720	920734	predicted RNA	87	331	0,032926	14
2964862	2964897	predicted RNA	229	69	0,032926	35

183197	183211	predicted RNA	531	159	0,033967	14
711451	711538	predicted RNA	8	32	0,033999	87
2632157	2632199	predicted RNA	99	28	0,03523	42
832332	832341	predicted RNA	205	56	0,03568	9
2104895	2104991	predicted RNA	213	481	0,036109	96
3219225	3219283	predicted RNA	316	109	0,036109	58
3306546	3306639	predicted RNA	24	88	0,038506	93
1230599	1230659	predicted RNA	147	46	0,039451	60
4278519	4278561	predicted RNA	18	70	0,039633	42
3372506	3372554	predicted RNA	126	38	0,040625	48
557315	557512	predicted RNA	72	178	0,04103	197
3417765	3417795	predicted RNA	10	35	0,041244	30
3972710	3972730	predicted RNA	66	19	0,041618	20
58011	58187	predicted RNA	196	79	0,041899	176
1642485	1642542	predicted RNA	37	10	0,042913	57
2409662	2409704	predicted RNA	190	59	0,042913	42
1666053	1666103	predicted RNA	74	247	0,043793	50
2864845	2865139	predicted RNA	121	49	0,043975	294
972503	972536	predicted RNA	35	10	0,044383	33
2317126	2317166	predicted RNA	53	191	0,044642	40
3989298	3989331	predicted RNA	22	80	0,045403	33
2505708	2505725	predicted RNA	18	65	0,045758	17
3998040	3998130	predicted RNA	49	14	0,045758	90
1696450	1696732	predicted RNA	18	56	0,046877	282
2073837	2073949	predicted RNA	745	325	0,047199	112
608545	608591	predicted RNA	539	209	0,047325	46
3855101	3855194	predicted RNA	53	16	0,047361	93
590974	590990	predicted RNA	121	34	0,047417	16
1720003	1720022	predicted RNA	24	88	0,04825	19
1321483	1321525	predicted RNA	165	51	0,048454	42
2739697	2739737	predicted RNA	163	50	0,04891	40
4384164	4384324	predicted RNA	88	33	0,04891	160
3302585	3303112	predicted RNA	42	91	0,049025	527
3323063	3323078	predicted RNA	67	19	0,049439	15
1806023	1806149	predicted RNA	98	34	0,049531	126
3894101	3894164	predicted RNA	70	21	0,049609	63

Supplementary Table S2: Differentially expressed sRNA between KZN and H37Rv strains (*q*<0.05)

Transcription Start	Transcription Stop	Product	Expression H37Rv	Expression KZN	qValue H37Rv vs KZN	Size
47208	47220	predicted RNA	64	0	0	12
82671	82711	predicted RNA	98	2	0	40
104708	104747	predicted RNA	96	0	0	39
148390	148429	predicted RNA	0	99	0	39
218653	218692	predicted RNA	0	55	0	39
223529	223561	predicted RNA	151	4	0	32
248933	249037	predicted RNA	64	0	0	104
314237	314278	predicted RNA	0	48	0	41
336311	336342	predicted RNA	0	45	0	31
415470	415501	predicted RNA	124	1	0	31
445235	445273	predicted RNA	0	94	0	38
475746	475781	predicted RNA	0	45	0	35
503310	503350	predicted RNA	0	98	0	40
508559	508579	predicted RNA	48	0	0	20
569835	569917	predicted RNA	0	48	0	82
586373	586386	predicted RNA	69	0	0	13
626444	626459	predicted RNA	72	0	0	15
700204	700227	predicted RNA	48	0	0	23
702770	702799	predicted RNA	0	53	0	29
717065	717107	predicted RNA	1	75	0	42
728368	728408	predicted RNA	0	40	0	40
736228	736246	predicted RNA	158	0	0	18
778934	778968	predicted RNA	0	74	0	34
808702	808726	predicted RNA	0	45	0	24
829168	829193	predicted RNA	0	45	0	25
833617	833655	predicted RNA	48	0	0	38
840880	840933	predicted RNA	0	40	0	53
855875	855916	predicted RNA	83	0	0	41
867393	867403	predicted RNA	0	47	0	10
901560	901620	predicted RNA	0	74	0	60
921141	921151	predicted RNA	0	45	0	10
982586	982627	predicted RNA	0	45	0	41
1002756	1002783	predicted RNA	0	40	0	27
1058195	1058252	predicted RNA	0	45	0	57
1076683	1076710	predicted RNA	79	0	0	27
1077875	1077915	predicted RNA	100	0	0	40
1127033	1127069	predicted RNA	91	0	0	36
1138913	1138943	predicted RNA	0	71	0	30
1146551	1146564	predicted RNA	70	0	0	13
1164378	1164417	predicted RNA	0	134	0	39
1201668	1201710	predicted RNA	0	100	0	42
1287045	1287059	predicted RNA	56	0	0	14

1308835	1308863	predicted RNA	0	51	0	28
1399247	1399285	predicted RNA	0	78	0	38
1404617	1404646	predicted RNA	0	45	0	29
1425973	1426012	predicted RNA	0	58	0	39
1426121	1426162	predicted RNA	0	65	0	41
1499196	1499215	predicted RNA	0	68	0	19
1547703	1547743	predicted RNA	0	81	0	40
1579587	1579603	predicted RNA	156	4	0	16
1622181	1622212	predicted RNA	0	45	0	31
1673306	1673336	predicted RNA	0	120	0	30
1692722	1692742	predicted RNA	131	0	0	20
1718703	1718724	predicted RNA	141	0	0	21
1784406	1784503	predicted RNA	26	0	0	97
1923697	1923731	predicted RNA	1	51	0	34
1986687	1986816	predicted RNA	82	0	0	129
1995966	1996001	predicted RNA	0	40	0	35
2002539	2002573	predicted RNA	56	0	0	34
2038690	2038732	predicted RNA	66	0	0	42
2048042	2048065	predicted RNA	67	0	0	23
2092183	2092226	predicted RNA	105	0	0	43
2157319	2157341	predicted RNA	0	67	0	22
2181914	2181924	predicted RNA	0	77	0	10
2223260	2223301	predicted RNA	1	65	0	41
2234910	2234964	predicted RNA	0	93	0	54
2263164	2263270	predicted RNA	0	25	0	106
2291944	2291969	predicted RNA	0	51	0	25
2372578	2372595	predicted RNA	0	51	0	17
2441749	2441801	predicted RNA	0	65	0	52
2449971	2449981	predicted RNA	133	0	0	10
2486217	2486232	predicted RNA	114	0	0	15
2506168	2506207	predicted RNA	0	62	0	39
2534026	2534046	predicted RNA	0	51	0	20
2541401	2541411	predicted RNA	0	60	0	10
2565289	2565319	predicted RNA	0	51	0	30
2574089	2574105	predicted RNA	0	40	0	16
2582334	2582365	predicted RNA	66	0	0	31
2614679	2614702	predicted RNA	0	65	0	23
2620031	2620075	predicted RNA	0	69	0	44
2705654	2705717	predicted RNA	0	39	0	63
2713815	2713841	predicted RNA	0	47	0	26
2734272	2734303	predicted RNA	0	40	0	31
2801195	2801241	predicted RNA	104	0	0	46
2976970	2976998	predicted RNA	0	50	0	28
3000066	3000110	predicted RNA	101	0	0	44

3025348	3025393	predicted RNA	99	0	0	45
3046631	3046663	predicted RNA	48	0	0	32
3074566	3074605	predicted RNA	89	0	0	39
3076826	3076844	predicted RNA	0	59	0	18
3095079	3095116	predicted RNA	48	0	0	37
3096885	3096921	predicted RNA	0	56	0	36
3098949	3098972	predicted RNA	82	0	0	23
3113484	3113510	predicted RNA	0	40	0	26
3119518	3119547	predicted RNA	0	40	0	29
3221729	3221768	predicted RNA	0	68	0	39
3282942	3283010	predicted RNA	48	0	0	68
3355098	3355108	predicted RNA	150	0	0	10
3378513	3378528	predicted RNA	0	71	0	15
3381008	3381021	predicted RNA	0	45	0	13
3457647	3457687	predicted RNA	0	45	0	40
3458168	3458207	predicted RNA	66	0	0	39
3487711	3487780	predicted RNA	0	82	0	69
3531136	3531156	predicted RNA	0	77	0	20
3547094	3547178	predicted RNA	0	13	0	84
3614414	3614453	predicted RNA	0	136	0	39
3633582	3633600	predicted RNA	0	40	0	18
3639406	3639418	predicted RNA	0	71	0	12
3682085	3682106	predicted RNA	0	45	0	21
3708491	3708510	predicted RNA	0	40	0	19
3720014	3720107	predicted RNA	0	34	0	93
3754439	3754463	predicted RNA	0	89	0	24
3805756	3805800	predicted RNA	61	0	0	44
3815179	3815206	predicted RNA	2	93	0	27
3926602	3926661	predicted RNA	66	0	0	59
3944950	3944989	predicted RNA	0	55	0	39
3945049	3945093	predicted RNA	0	75	0	44
3945153	3945198	predicted RNA	0	47	0	45
3964546	3964584	predicted RNA	0	54	0	38
3965499	3965556	predicted RNA	0	30	0	57
3965644	3965683	predicted RNA	0	56	0	39
3972710	3972730	predicted RNA	66	0	0	20
4025897	4025936	predicted RNA	0	40	0	39
4054231	4054276	predicted RNA	265	3	0	45
4062522	4062570	predicted RNA	56	0	0	48
4080740	4080749	predicted RNA	0	51	0	9
4095163	4095178	predicted RNA	90	0	0	15
4129983	4129999	predicted RNA	0	51	0	16
4149259	4149268	predicted RNA	0	62	0	9
4156921	4156962	predicted RNA	0	57	0	41

4166938	4166955	predicted RNA	2	71	0	17
4180807	4180849	predicted RNA	0	45	0	42
4215954	4215989	predicted RNA	0	74	0	35
4216242	4216255	predicted RNA	75	0	0	13
4243326	4243348	predicted RNA	0	64	0	22
4278363	4278403	predicted RNA	0	56	0	40
4312867	4312908	predicted RNA	0	45	0	41
4317634	4317691	predicted RNA	72	0	0	57
51816	51827	predicted RNA	80	0	2,1E-269	11
591018	591033	predicted RNA	0	51	1E-257	15
2630330	2630386	predicted RNA	184	6	2,6E-250	56
2135912	2135936	predicted RNA	59	2	3E-196	24
1547580	1547622	predicted RNA	87	3	1,8E-173	42
1409413	1409441	predicted RNA	111	5	1,5E-139	28
3873587	3873596	predicted RNA	66	0	1,9E-139	9
4043120	4043130	predicted RNA	0	51	2E-135	10
1690381	1690394	predicted RNA	0	40	1,4E-131	13
3135665	3135788	predicted RNA	2	50	6,9E-130	123
463395	463411	predicted RNA	4	79	2,1E-127	16
2870381	2870421	predicted RNA	113	5	3,9E-116	40
1238201	1238210	predicted RNA	0	45	4,09E-92	9
1951759	1951768	predicted RNA	48	0	7,39E-81	9
2694981	2694990	predicted RNA	48	0	7,39E-81	9
2716337	2716384	predicted RNA	62	3	1,49E-72	47
978920	978936	predicted RNA	173	10	2,06E-67	16
3080486	3080590	predicted RNA	191	10	2,16E-63	104
2850883	2850905	predicted RNA	96	6	1,51E-58	22
1989722	1989763	predicted RNA	96	6	7,38E-55	41
2153097	2153154	predicted RNA	3	57	1,26E-52	57
456194	456268	predicted RNA	485	32	5,37E-48	74
119718	119757	predicted RNA	80	5	8,27E-43	39
133771	133947	predicted RNA	51	519	1,64E-39	176
1176865	1176899	predicted RNA	3	45	2,37E-38	34
993843	993855	predicted RNA	7	80	9,07E-34	12
154141	154229	predicted RNA	81	6	1,03E-33	88
3007073	3007199	predicted RNA	7	88	8,84E-31	126
3843114	3843215	predicted RNA	129	10	2,17E-30	101
4235480	4235528	predicted RNA	152	12	9,09E-29	48
2326885	2326932	predicted RNA	4	43	2,66E-25	47
4327683	4327726	predicted RNA	281	24	1,38E-22	43
876576	876617	predicted RNA	116	10	2,43E-22	41
1728416	1728958	predicted RNA	662	105	4,2E-22	542
29209	29244	predicted RNA	164	15	1,16E-20	35
296622	296796	predicted RNA	120	11	5,83E-19	174

1165536	1165626	predicted RNA	77	621	7,37E-19	90
2876502	2876527	predicted RNA	369	37	4,91E-16	25
1933976	1934154	predicted RNA	1082	195	2,27E-15	178
1673402	1673445	predicted RNA	217	22	5,14E-15	43
978260	978341	predicted RNA	19	181	2,4E-14	81
4299784	4299794	predicted RNA	1396	150	4,13E-14	10
4137346	4137383	predicted RNA	20	181	5,3E-14	37
2156658	2156712	predicted RNA	8	73	7,77E-14	54
4338700	4339009	predicted RNA	10	85	9,32E-14	309
1241578	1241605	predicted RNA	112	925	9,93E-14	27
1166822	1166932	predicted RNA	4191	794	2,5E-13	110
3162169	3162277	predicted RNA	5	50	5,42E-13	108
476662	476681	predicted RNA	18	153	7,27E-13	19
999303	999357	predicted RNA	108	12	4,41E-12	54
1051503	1051547	predicted RNA	81	9	1,52E-11	44
1236178	1236189	predicted RNA	9	58	8,3E-11	11
4069234	4069252	predicted RNA	132	16	8,5E-11	18
742635	742697	predicted RNA	163	20	1,97E-10	62
2763831	2763897	predicted RNA	318	42	7,29E-10	66
3969345	3969394	predicted RNA	11	89	8,03E-10	49
3430318	3430383	predicted RNA	4	33	8,28E-10	65
1783236	1783315	predicted RNA	88	11	2,52E-09	79
1135471	1135504	predicted RNA	6	45	7,17E-09	33
2562468	2562486	predicted RNA	7	51	7,17E-09	18
862385	862412	predicted RNA	148	20	1,37E-08	27
4093744	4093809	predicted RNA	2893	680	2,44E-08	65
3894572	3894604	predicted RNA	15	108	2,96E-08	32
186662	186757	predicted RNA	79	10	3,63E-08	95
4190440	4190461	predicted RNA	16	109	4,3E-08	21
3827139	3827168	predicted RNA	217	29	6,7E-08	29
3964257	3964350	predicted RNA	77	10	6,98E-08	93
3552585	3552599	predicted RNA	463	64	8,32E-08	14
4340206	4340223	predicted RNA	108	16	3,61E-07	17
3393952	3393997	predicted RNA	13	86	7,66E-07	45
3499925	3499939	predicted RNA	18	118	8,12E-07	14
3186983	3187035	predicted RNA	82	461	1,62E-06	52
3388043	3388078	predicted RNA	354	54	1,64E-06	35
3944797	3944838	predicted RNA	15	100	1,83E-06	41
2074649	2074755	predicted RNA	84	12	2,78E-06	106
3594233	3594448	predicted RNA	22	121	3,99E-06	215
1300130	1300308	predicted RNA	1740	470	5,2E-06	178
1271150	1271160	predicted RNA	245	38	6,31E-06	10
3643026	3643168	predicted RNA	7	46	7,03E-06	142
993524	993685	predicted RNA	70	11	7,12E-06	161

3818208	3818221	predicted RNA	26	152	1,99E-05	13
3032511	3032529	predicted RNA	156	25	2,65E-05	18
1320007	1320040	predicted RNA	256	41	2,8E-05	33
3919380	3919398	predicted RNA	16	95	2,97E-05	18
838056	838453	predicted RNA	704	203	3,16E-05	397
3822422	3822431	predicted RNA	127	22	3,53E-05	9
2565178	2565221	predicted RNA	136	22	4,64E-05	43
3482724	3482772	predicted RNA	161	27	6,4E-05	48
3893900	3894213	predicted RNA	55	186	7,83E-05	313
2775227	2775248	predicted RNA	12	69	8,62E-05	21
4231417	4231497	predicted RNA	118	20	9,14E-05	80
1227590	1227600	predicted RNA	124	22	0,000102	10
3418702	3418722	predicted RNA	130	22	0,000114	20
289043	289062	predicted RNA	109	19	0,000141	19
3022383	3022413	predicted RNA	109	19	0,000141	30
608705	608748	predicted RNA	89	15	0,000153	43
1243618	1243711	predicted RNA	226	45	0,000175	93
1617581	1617842	predicted RNA	9	51	0,000183	261
2934961	2935018	predicted RNA	16	88	0,000219	57
1345186	1345235	predicted RNA	108	19	0,000231	49
168671	168701	predicted RNA	17	92	0,000231	30
4014221	4014246	predicted RNA	84	15	0,000252	25
3837676	3837716	predicted RNA	10	51	0,000282	40
2330155	2330184	predicted RNA	14	73	0,000319	29
1060582	1060627	predicted RNA	102	18	0,000348	45
3551073	3551230	predicted RNA	65	244	0,000367	157
3953515	3953578	predicted RNA	254	50	0,000488	63
4348903	4349004	predicted RNA	17	90	0,000488	101
2393650	2393843	predicted RNA	23	105	0,000558	193
4314256	4314353	predicted RNA	131	25	0,000595	97
2226176	2226251	predicted RNA	222	45	0,0006	75
4162441	4162480	predicted RNA	58	11	0,000632	39
1537719	1537739	predicted RNA	384	71	0,000647	20
3123047	3123627	predicted RNA	44	123	0,000647	580
3173061	3173112	predicted RNA	17	88	0,000664	51
2573758	2573822	predicted RNA	71	13	0,000708	64
3041519	3041558	predicted RNA	81	15	0,00092	39
382419	382469	predicted RNA	24	122	0,000965	50
4036866	4036906	predicted RNA	207	39	0,001106	40
3427220	3427239	predicted RNA	23	108	0,001117	19
4197774	4197805	predicted RNA	211	40	0,00119	31
944821	944891	predicted RNA	146	29	0,001353	70
669749	669766	predicted RNA	227	42	0,001365	17
3119747	3120541	predicted RNA	4	20	0,001387	794

1418960	1419015	predicted RNA	693	197	0,001389	55
577342	577564	predicted RNA	31	124	0,001428	222
1616356	1616966	predicted RNA	50	13	0,001428	610
1782592	1782764	predicted RNA	353	107	0,001532	172
2495410	2495424	predicted RNA	18	89	0,001548	14
1341318	1341335	predicted RNA	274	53	0,001728	17
1594030	1594047	predicted RNA	129	25	0,001793	17
1206483	1206494	predicted RNA	471	92	0,00188	11
2866423	2866449	predicted RNA	18	88	0,00199	26
2614074	2614113	predicted RNA	628	155	0,002239	39
3726047	3726280	predicted RNA	1695	531	0,002408	233
455397	455442	predicted RNA	128	25	0,002582	45
4323476	4323558	predicted RNA	171	37	0,002754	82
3030960	3031049	predicted RNA	11	56	0,002837	89
1274773	1274819	predicted RNA	3100	1092	0,003306	46
183197	183211	predicted RNA	531	108	0,00331	14
618287	618307	predicted RNA	18	83	0,003528	20
3174654	3174705	predicted RNA	207	44	0,003551	51
3585993	3586269	predicted RNA	406	953	0,003567	276
967821	967856	predicted RNA	225	46	0,003833	35
2712887	2712898	predicted RNA	103	22	0,004256	11
1825936	1826001	predicted RNA	10	50	0,004265	65
3554057	3554066	predicted RNA	61	283	0,004659	9
876394	876500	predicted RNA	13	63	0,004659	106
4067547	4067601	predicted RNA	405	103	0,00513	54
3707735	3707755	predicted RNA	50	233	0,005131	20
87131	87202	predicted RNA	133	29	0,005208	71
3907601	3907720	predicted RNA	12647	2991	0,005308	119
1445379	1445497	predicted RNA	289	88	0,00543	118
779491	779545	predicted RNA	192	43	0,006003	54
1417354	1417663	predicted RNA	132	42	0,00689	309
3769673	3769684	predicted RNA	113	25	0,007109	11
2897894	2897936	predicted RNA	80	17	0,007869	42
1090180	1090235	predicted RNA	122	26	0,008288	55
749970	750002	predicted RNA	115	24	0,008336	32
1733557	1733614	predicted RNA	153	34	0,008875	57
287118	287163	predicted RNA	394	100	0,009092	45
2627001	2627047	predicted RNA	110	24	0,0092	46
4041538	4041601	predicted RNA	62	13	0,00944	63
2248463	2248570	predicted RNA	19	81	0,009768	107
277853	277871	predicted RNA	199	42	0,010069	18
2531982	2532222	predicted RNA	305	109	0,010069	240
1325684	1325760	predicted RNA	218	56	0,011664	76
2224170	2224227	predicted RNA	123	27	0,01205	57

2446995	2447043	predicted RNA	83	18	0,012058	48
1518619	1518658	predicted RNA	103	22	0,012097	39
72220	72270	predicted RNA	28	122	0,012297	50
2651988	2652034	predicted RNA	21	91	0,012303	46
612910	613040	predicted RNA	6092	1751	0,012936	130
1533640	1533685	predicted RNA	164	37	0,013082	45
3409461	3409495	predicted RNA	138	31	0,013295	34
2991127	2991193	predicted RNA	134	421	0,013402	66
3291424	3291512	predicted RNA	10	42	0,013462	88
657474	657490	predicted RNA	99	22	0,013578	16
2639566	2639670	predicted RNA	61	13	0,013578	104
753371	753456	predicted RNA	11	49	0,014247	85
419609	419788	predicted RNA	661	251	0,014365	179
1912937	1912980	predicted RNA	66	15	0,0149	43
2089526	2089679	predicted RNA	85	21	0,015341	153
606452	606553	predicted RNA	160	42	0,015572	101
678346	678370	predicted RNA	65	15	0,01729	24
330326	330421	predicted RNA	641	233	0,017308	95
14882	14913	predicted RNA	104	23	0,01741	31
3755648	3755746	predicted RNA	63	14	0,017593	98
3786453	3786473	predicted RNA	27	113	0,017593	20
965539	965985	predicted RNA	36	93	0,017694	446
3082827	3082918	predicted RNA	56	13	0,017895	91
3193382	3193402	predicted RNA	97	22	0,018061	20
2558824	2558860	predicted RNA	13	55	0,019054	36
1518724	1518733	predicted RNA	14	52	0,019212	9
48101	48141	predicted RNA	86	19	0,019329	40
2238106	2238148	predicted RNA	74	17	0,020497	42
3153006	3153046	predicted RNA	247	61	0,020577	40
3907187	3907389	predicted RNA	70	18	0,020781	202
3133590	3133600	predicted RNA	56	15	0,020811	10
1418363	1418400	predicted RNA	20	85	0,020972	37
2025081	2025145	predicted RNA	65	15	0,021369	64
2884460	2884620	predicted RNA	343	125	0,02166	160
3820583	3820766	predicted RNA	88	222	0,021745	183
4305899	4305928	predicted RNA	99	23	0,021982	29
3232644	3232880	predicted RNA	73	180	0,022298	236
3379346	3379385	predicted RNA	87	20	0,022298	39
4249989	4250051	predicted RNA	68	16	0,022458	62
4306990	4307025	predicted RNA	130	30	0,022559	35
43366	43409	predicted RNA	294	78	0,02295	43
34033	34294	predicted RNA	125	43	0,02367	261
2430100	2430128	predicted RNA	149	35	0,024682	28
2934180	2934207	predicted RNA	110	25	0,025007	27

219366	219483	predicted RNA	76	19	0,02506	117
671156	671168	predicted RNA	146	35	0,025752	12
4317458	4317506	predicted RNA	212	54	0,025816	48
3421602	3421681	predicted RNA	220	63	0,027066	79
2545642	2545663	predicted RNA	566	152	0,028126	21
4400986	4401046	predicted RNA	1094	419	0,028126	60
2540035	2540113	predicted RNA	115	29	0,028157	78
4046359	4046383	predicted RNA	60	15	0,028869	24
1346912	1346925	predicted RNA	29	110	0,028893	13
2047364	2047644	predicted RNA	193	72	0,028952	280
212265	212274	predicted RNA	86	22	0,029314	9
2225246	2225405	predicted RNA	297	109	0,029583	159
902099	902113	predicted RNA	103	25	0,031059	14
2569018	2569059	predicted RNA	12	46	0,031091	41
3491657	3491802	predicted RNA	22	79	0,032447	145
2566561	2566776	predicted RNA	147	52	0,033526	215
2752217	2752255	predicted RNA	94	22	0,034197	38
3029141	3029181	predicted RNA	92	22	0,034345	40
141111	141151	predicted RNA	82	20	0,03537	40
2217655	2217666	predicted RNA	75	20	0,03537	11
3097662	3097715	predicted RNA	60	212	0,03537	53
3569043	3569082	predicted RNA	51	193	0,037359	39
3148352	3148362	predicted RNA	24	88	0,037804	10
4117398	4117435	predicted RNA	86	21	0,039347	37
1519992	1520006	predicted RNA	24	89	0,0394	14
29630	29681	predicted RNA	371	115	0,040278	51
264024	264063	predicted RNA	424	128	0,04109	39
927796	927806	predicted RNA	84	22	0,041179	10
4078648	4078667	predicted RNA	24	88	0,041413	19
3307504	3307589	predicted RNA	31	112	0,042066	85
2915748	2915855	predicted RNA	46	148	0,043585	107
2456810	2456907	predicted RNA	309	113	0,045036	97
388551	388570	predicted RNA	24	86	0,045374	19
1981088	1981136	predicted RNA	37	139	0,046153	48
2498617	2498686	predicted RNA	27	100	0,046153	69
2795230	2795310	predicted RNA	44	148	0,046931	80
1617393	1617435	predicted RNA	23	87	0,047273	42
2957555	2957581	predicted RNA	20	78	0,047475	26
2343983	2344001	predicted RNA	59	223	0,047927	18
580594	580811	predicted RNA	350	142	0,049209	217
2740410	2740710	predicted RNA	113	225	0,049563	300
3073067	3073100	predicted RNA	57	209	0,049603	33

Supplementary Table S3: The 36 common differentially expressed sRNAs in clinical strains of *M. tuberculosis*.

	KZN	Beijing	H37Rv
Transcript_32	4	15	151
Transcript_15	0	16	72
Transcript_34	74	67	0
Transcript_36	0	19	91
Transcript_16	4	35	156
Transcript_20	0	20	131
Transcript_21	0	11	141
Transcript_129	0	6	82
Transcript_46	0	7	104
Transcript_39	0	14	89
Transcript_44	0	15	61
Transcript_20	0	19	66
Transcript_11	0	2	80
Transcript_42	3	6	87
Transcript_9	0	0	48
Transcript_48	12	3	152
Transcript_41	10	20	116
Transcript_43	22	32	217
Transcript_19	153	165	18
Transcript_44	9	13	81
Transcript_18	16	19	132
Transcript_79	11	9	88
Transcript_95	10	6	79
Transcript_33	41	9	256
Transcript_18	95	62	16
Transcript_19	19	6	109
Transcript_63	50	79	254
Transcript_172	107	72	353
Transcript_14	0	159	531
Transcript_130	0	1770	6092
Transcript_179	0	278	661
Transcript_42	0	1	74
Transcript_24	0	6	60
Transcript_14	0	26	103
Transcript_215	0	51	147
Transcript_37	0	10	86
